# Metabolomics as a potential tool for the diagnosis of growth hormone deficiency (GHD): a review

**DOI:** 10.20945/2359-3997000000300

**Published:** 2020-10-21

**Authors:** Breno Sena De San-Martin, Vinícius Guimarães Ferreira, Mariana Rechia Bitencourt, Paulo Cesar Gonçalves Pereira, Emanuel Carrilho, Nilson Antônio de Assunção, Luciani Renata Silveira de Carvalho

**Affiliations:** 1 Universidade Federal de São Paulo Escola Paulista de Medicina São Paulo SP Brasil Escola Paulista de Medicina da Universidade Federal de São Paulo (EPM-UNIFESP), São Paulo, SP, Brasil; 2 Universidade de São Paulo Instituto de Química de São Carlos São Carlos SP Brasil Instituto de Química de São Carlos da Universidade de São Paulo (IQSC-USP), São Carlos, SP, Brasil; 3 Instituto Nacional de Ciência e Tecnologia de Bioanalítica Campinas SP Brasil Instituto Nacional de Ciência e Tecnologia de Bioanalítica – INCTBio, Campinas, SP, Brasil; 4 Universidade de São Paulo Faculdade de Medicina Disciplina de Endocrinologia São Paulo SP Brasil Unidade de Endocrinologia do Desenvolvimento, Laboratório de Hormônios e Genética Molecular LIM42, Disciplina de Endocrinologia, Faculdade de Medicina da Universidade de São Paulo (FMUSP), São Paulo, SP, Brasil; 5 Universidade Federal de São Paulo Instituto de Ciências Ambientais, Químicas e Farmacêuticas Departamento de Química Diadema SP Brasil Departamento de Química, Instituto de Ciências Ambientais, Químicas e Farmacêuticas, Universidade Federal de São Paulo, Diadema, SP, Brasil

**Keywords:** Metabolomics, GHD, metabolites, pathophysiology, translational

## Abstract

Metabolomics uses several analytical tools to identify the chemical diversity of metabolites present in organisms. These metabolites are low molecular weight molecules (<1500 Da) classified as a final or intermediary product of metabolic processes. The application of this omics technology has become prominent in inferring physiological conditions through reporting on the phenotypic state; therefore, the introduction of metabolomics into clinical studies has been growing in recent years due to its efficiency in discriminating pathophysiological states. Regarding endocrine diseases, there is a great interest in verifying comprehensive and individualized physiological scenarios, in particular for growth hormone deficiency (GHD). The current GHD diagnostic tests are laborious and invasive and there is no exam with ideal reproducibility and sensitivity for diagnosis neither standard GH cut-off point. Therefore, this review was focussed on articles that applied metabolomics in the search for new biomarkers for GHD. The present work shows that the applications of metabolomics in GHD are still limited, since the little complementarily of analytical techniques, a low number of samples, GHD combined to other deficiencies, and idiopathic diagnosis shows a lack of progress. The results of the research are relevant and similar; however, their results do not provide an application for clinical practice due to the lack of multidisciplinary actions that would be needed to mediate the translation of the knowledge produced in the laboratory, if transferred to the medical setting.

## INTRODUCTION

Large-scale molecular studies have been highlighted since the sequencing of the human genome in the late twentieth century, when it was believed that knowledge of the complete sequence would be sufficient to completely understand all the biological processes ( 1 ). However, researchers have come across an even larger issue, to understand the mechanism and changes in genome functioning at different stages of development and environmental conditions ( 2 ). The central dogma of molecular biology, postulated by Crick in 1958 ( 3 ), proposed that the flow of information through the cell signaling mechanism is from DNA to proteins, and that there is no flow of information from proteins to proteins, or from proteins to DNA. Although it remains valid even today, chemical biology has found a further piece for this puzzle, the small molecules. With the small molecules playing an important role in cell mechanisms, genomics, e.g., DNA sequencing, cannot be seen as the single tool that fulfills the gap between DNA and cell phenotypes. Consequently, it is in the “post-genomics” era, with the new “omics” sciences (e.g., transcriptomics, proteomics, lipidomics, and metabolomics) that scientists aim to accomplish their understanding of the link between genetics and the phenotype or physiological condition of an organism, and finally complete the “central dogma” puzzle comprising thousands of pieces ( 4 ).

The so-called “omics” sciences came from the Greek suffix “ome”, which can be understood as “set of a substance”, hence, proteomics may be understood as the study of the “set of proteins”; lipidomics as the study of the “set of lipids”, and metabolomics as the study of the “set of metabolites”. Amongst the omics sciences, the emerging field of metabolomics has stood out for capturing and applying various analytical approaches in order to identify and or quantify metabolites present in biological matrices, and it is particularly interesting due the metabolites being the end products of cellular processes; thus, it is the closest link between biochemical reactions and physiological conditions *, e.g.,* cancer, birth diseases, and immunologic disorders ( 5 – 8 ).

**Figure 1:**
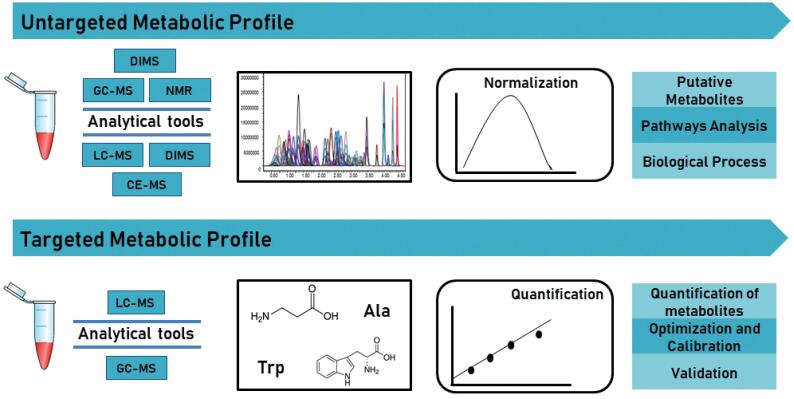
Targeted versus untargeted metabolomics approaches.

Metabolites comprise an enormous chemical diversity ( *e.g* ., sugars, amino acids, organic acids, nucleotides, acylcarnitines, and lipids) and present several differences in their concentrations in the biological system. Therefore, in the current technological conjuncture, the use of more than one technique is indispensable in order to analyze the highest portion of the metabolome, in its concentration and identity, and to seek an efficient construction of metabolomic profiles. Metabolomics is divided into two main approaches: target (nonglobal) metabolomics, and untargeted (global) metabolomics ( Figure 1 ); both seek to find differences in the biological system's metabolome, but target metabolomics is carried out by focusing on one, or a few specific metabolites, while untargeted metabolomics focuses on a broad variety of metabolites ( 9 ).

**Figure 2:**
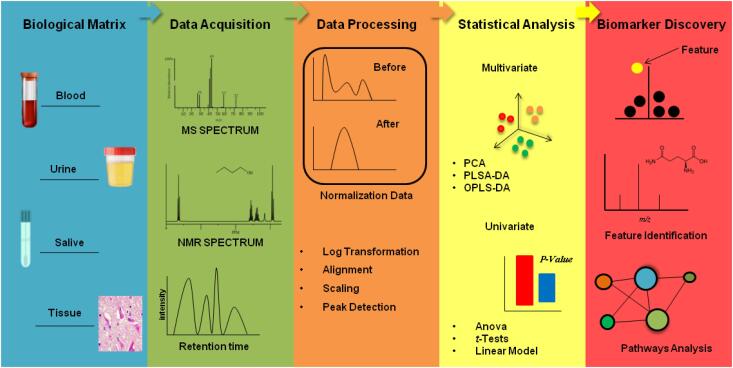
Five steps in the metabolomics workflow.

The increasing application of metabolomics in the clinical/medical fields is due to the success of obtaining a broad perspective of the physiological state of organisms through different biological matrices, such as cells, tissues, or biofluids, leading to rapid, sensitive, and less invasive analyses ( 10 – 12 ). Recent research has been directed at elucidating the responses and changes promoted by genetic, epigenetic, protein modifications, and also external factors such as nutrition, physical activity, and environmental exposure, which are correlated in order to understand the pathophysiology, molecular mechanisms, and identification of possible biomarkers that come out in the metabolism under different conditions (i.e.,disease, nutrition, environmental exposure) ( 2 ). The workflow in metabolomics, besides small differences from lab to lab, is well established and is quite simple, and will be discussed later in this review ( Figure 2 ) ( 13 ).

Due to the logical and scientific capacity of this workflow, there is a large set of questions that may be answered by metabolomics and a large number of diseases that can be studied by this scientific approach. In the past few years, several papers have been published that have analyzed the complete metabolism of diseases such as cancer, cardiovascular events, and endocrine disorders ( 14 – 16 ). The former is particularly interesting since it incorporates a set of common disorders, such as diabetes type 1 and 2, polycystic ovary syndrome, obesity, and thyroid disorders that can influence several metabolic pathways and affect one of the most basic processes in human development, the ability to grow ( 14 , 16 ). Due to the high capacity available to research the human metabolome, there is a growing interest in applying metabolomics to understanding changes in the endocrine system affected by conditions of abnormal GH secretion as well as doping with rhGH.

Growth in humans is dependent on the growth hormone (GH), which can be broadly conceptualized as a protein and/or 191-amino-acid polypeptide hormone synthesized by the pituitary gland ( 17 , 18 ). Insufficient production of GH generates the disease known as GHD and is implicated in growth problems in childhood, which presents as short stature. Currently, the importance of the hormone has also been observed in adulthood, especially for lipid profile, body composition, and bone mass, as well as possible benefits in cognition ( 19 ).

The diagnosis of GHD requires, in case of clinical suspicion, an extensive evaluation that goes through laboratory tests and imaging examinations. IGF-1 (insulin-like growth factor 1) and IGFBP-3 (insulin-like growth factor binding protein 3) are indirect markers of growth hormone action. Another factor that contributes to the difficulty in diagnosing growth hormone deficiency is the stage of life in which tests are performed and what are more appropriated for each period. Clonidine stimulous test is indicated for children with short stature while it is not considered adequate for adult, once the gold standard test in adult is insulin hypoglycemia test (ITT). In childhood IGF1 and IGFBP3 have good specificity but low sensitivity ( 20 ); hence, regardless of the stage of life in which GHD is investigated it is necessary to expose the patient to tests capable of inducing maximum GH secretion by the pituitary gland. It is documented that adult patients with adquired GHD can have normal IGF1 for age and once under stimulous test, the response can be absent confirming GHD ( 21 – 24 ). Another issue for diagnosis is the GH cut off level that is variable with methodology and population even children or adult patient, and sometimes, they are not reproducible even in the same individual, specially children ( 20 ). Despite this arsenal of tests to diagnose GHD and new GH monoclonal antibody assays, there is no new data demonstrating the normal range for stimulated GH levels ( 25 ). This whole context leads to difficult test interpretations and none of the tests used alone are proven efficient or sufficient in accurately determining the diagnosis due to the lack of uniformity and reproduction in the results ( 26 – 28 ).

GHD in children and adults have different diagnosis procedures, different treatment regimens and efficacy control ( 19 , 23 ). In children the most important efficacy control is adequate growth. In adults, The current clinical tools recommended for monitoring recombinant human growth hormone (rhGH) replacement, include anthropometric measurements of weight and BMI, serum IGF-1 measurements, lipid profile, and glycemic control assessment such as glycated hemoglobin; however, pre-existing metabolic dysfunctions at the start of rhGH treatment are confounding factors in measuring the benefits of replacement therapy. In addition, IGF-1 alone is known not to be a reliable marker of GHD diagnosis in adults, emphasizing the difficulty in diagnosing GHD ( 21 ).

Nowadays, the best treatment for adults with GHD consists of replacement therapy with rhGH. This treatment approach is known to decrease cardiovascular risk factors, while GH cessation reverses most of these factors. However, it is important to highlight that metabolomics potential to explain the changes is still little understood in relation to GH replacement associated with increased risk of cardiovascular disease. Treatment with GH itself can lead to insulin resistance, which also probably influences cardiovascular health status ( 29 ).

Notwithstanding, the use of metabolomics in GH deficiency is an important approach to reach a more general, rather than the reductionist profile of the pathophysiology ( 26 ). In this context, the presente paper intends to ascertain the adoption of metabolomics in understanding the pathophysiology of GHD using a systematic review. It aims to contextualize the technique of metabolomics succinctly, and explore how it is significantly contributing to the advancement of clinical research in GHD.

## LITERATURE SEARCH

The present review was based on a process of systematic review and meta-analysis ( 30 ). We searched PubMed with no date restrictions until February 2020. The search strategy used Boolean logic, which is an algorithm used in the PubMed platform focused on advanced searches. The terms used were: ‘metabolomics’ (All fields) OR ‘lipidomics’ (All fields) AND ‘growth hormone deficiency’ (All fields) OR ‘GHD’ (All fields). Additional studies were found by cross-checking references.

The inclusion and exclusion criteria for articles were as follow: for inclusion, any study that used metabolomics/lipidomics in biological samples from patients diagnosed with growth hormone deficiency. For exclusion, articles with unclear data and without an affirmed omics approach. The algorithm of Boolean logic in the PubMed platform revealed only three relevant articles used in metabolomics studies of patients with GHD. Table 1 shows the clinical conditions, sample type, analytical tool, cohort, and altered biochemical pathways of these studies.

**Table 1:** Relevant articles founded with the Boolean logic algorithm in the PubMed platform.

Clinicaldata	Sample type	Analytical tool	Cohort	Pathways	Ref
To compare the serum metabolome between GHD patients and healthy controls, and identification of potential markers for diagnosis and/or for individual GH dosing	Serum	GC-MS	10	Lipid and protein metabolism	[( 26 )]
A 17 year old female adolescent with severe GHD secondary to PIT-1 gene mutation. The subject was subsequently followed for 5 years with and without GH therapy	Urine	NMR	1	Beta-oxidation; TCA cycle	[( 27 )]
All patients with short stature were confirmed to be due to the endocrine disorders, especially GH deficiency (GHD)	Serum	NMR	45	Glucose; amino acids	[( 59 )]

GC-MS: gas chromatography mass spectrometry; NMR: nuclear magnetic resonance; TCA cycle: tricarboxylic acid cycle or Krebs cycle.

### Workflow in metabolomics study

The metabolomics workflow contains fundamental steps to achieve efficient results. In the untargeted approach, there is still no prior knowledge of metabolites that will be decisive in differentiating between groups' control (healthy) and case (disease) ( 31 ). Figure 2 shows the five essential steps in metabolomics studies, according to the protocols found in the literature: 1) The biological matrix is essential to extract metabolites correlated with the biological question. 2) The acquisition of data by analytical techniques should also be considered. The effectiveness and specificity of the tool should help and/or facilitate the acquisition of information about the chosen biological sample. 3) Data processing is inherent for treating large-scale molecular data. The data are obtained and subjected to data standardization processes to continue statistical analyses. 4) Mathematical and statistical models from chemometrics aim to understand the chemical data information extracted from the biological matrix. Therefore, the application of multivariate and univariate analyses are employed as filters to reduce the large number of features identified. 5) After filtering, prominent metabolites are determined and correlated with the pathophysiology. Putative identification of the main metabolites leads to the construction, through algorithms, of biochemical pathways inherent to these compounds.

### Biofluids

The very first step in a metabolomics study is to define which are the best biofluid samples to answer the main question. The diversity of biological matrices in metabolomics that are found in publications are numerous; saliva, urine, tissue, cerebrospinal fluid and sweat have all been reported ( 32 – 34 ). It is important to note that several compounds can be diffused in different fluids, for example, salivary ducts and blood circulation, and so there is a need to consider their integration and move to multi-biofluidic analyses ( 35 , 36 ). The physiological complexity of humans, and the genetic variation and chemical diversity of metabolites make it necessary to apply different analytical techniques and complementary approaches in the efficient creation of a metabolomic profile ( 10 ).

Blood is known to be the richest biofluid in GHD pathophysiological information; in the field of metabolomics and proteomics it aims to use less non-invasive biofluids that are rich in biochemical information as blood ( 37 ). The integration of data from omics technologies using the multi-fluid system appears to be in the near future. It will be possible to use more practical and non-invasive biofluids, such as saliva or urine, using different analytical tools ( 10 , 38 ).

### Analytical tools

Once the best biofluidic sample has been defined, it is essential to look for the best analytical tool to perform the analysis of this sample. In this step, the researchers must question if there are necessary complex sample preparation protocols, such as derivatization (i.e., changing the physical–chemical state) ( 39 ), or whether just a few steps extraction is enough for a broad analysis. Indeed, many scientists believe that the less the sample is handled, the better ( 40 ). However, in order to select the best sample preparation protocol, the researchers must first define what analytical techniques are most recommended for their research.

The most commonly used analytical tools in the field of bioanalytical applied metabolomics are nuclear magnetic resonance (NMR) and mass spectrometry (MS), whose relative advantages and disadvantages must be weighed up to obtain the required results. Many of the barriers found in NMR and MS techniques can be solved by coupling them with other well-known analytical techniques, including chromatography and capillary electrophoresis techniques, to complement the identification of metabolites ( 41 , 42 ).

The use of magnetic resonance spectroscopy has well-defined protocols for the acquisition and characterization of metabolites ( 42 ). Compared to MS, NMR is not as sensitive, however, it is quantifiable, reproducible, and does not need to fragment molecules, allowing successive analyses of the same sample. MS is a highly sensitive technique used for metabolite determination and quantification, which allows for the analysis of both known and unknown molecules. An inherent factor in MS are the hyphenated mass spectrometer separation techniques that, even though they are standardized and have high analytical power, have disadvantages in making analyses slow and expensive ( 42 ).

Chromatography is defined as a physicochemical substance separation technique based on the polarity principle ( 43 ). The counterpoint of using each analytical technique is balanced during the experimental design; gas chromatography coupled to mass spectrometry (CG-MS) efficiently separates thermally stable and/or volatile nonpolar metabolites ( 44 ), however liquid chromatography coupled to mass spectrometry (LC-MS) is more efficient in separating sugars, amino acids, vitamins, biogenic amines, carboxylic acids, and nucleotides ( 9 , 45 ). Capillary electrophoresis, coupled to mass spectrometry (CE-MS), has gained prominence in the past decade in the field because it is faster, has higher efficiency, versatility and lower cost relative to others. The use of CE-MS in the bioanalytical field is still recent and is an auxiliary tool in the characterization of polar metabolites, however, when compared to LC-MS, CE-MS presents much lower reproducibility and a less sophisticated hardware ( 41 ).

In recent years, with the development of high-resolution mass spectrometers, there have been significant increases in research using the direct infusion mass spectrometry (DIMS) method. Using DIMS, the use of separation techniques ( *e.g.,* liquid chromatography and capillary electrophoresis), become obsolete in the first instance, while the biological samples are injected directly into the ionization source of MS. This process is still recent, however, but promises to have advantages in reducing the acquisition time of spectral data, solvent use and energy spent ( 46 , 47 ).

For the study of GHD focusing on the lipid class, the most suitable approach would be to use DIMS using high-resolution mass spectrometers. The stereochemical structures of lipids (isobars) are not well elucidated with the aid of LC-MS; therefore, the use of high analytical resolution detectors would lead to more detailed results on the lipid compounds ( 48 ).

### Statistical analyses

In order to analyze the huge data set from the multiple analytical tools available, metabolomic studies require the use of chemometrics, which is the use of mathematical and statistical models to extract the most relevant information from the data set ( 49 ). The most popular multivariate methods used in metabolomics are partial component analysis (PCA), partial least square discriminant analysis (PLS-DA), and its orthogonal variant known as orthogonal partial least square discriminant analysis (OPLS-DA). The multivariate analysis enables the analysts to identify variables ( *i.e* ., compounds) of interest in the large amount of data, which would require an enormous amount of time to be done univariately. However, the analysis by PCA, in the best scenario, permits the user to separate the differentiated compounds without insertion of information about the groups ( *i.e.,* disease vs. control); it is called an unsupervised analysis, and is commonly done as the very first data analysis in a metabolomic study. On the other hand, PLS-DA and OPLS-DA allow the use of group information and are called supervised data analysis ( 50 ). Univariate analyses ( *i.e.,* ANOVA, t-tests) are also crucial methods, however, they are mainly used after the multivariate analysis, for filtering the metabolites that were shown to be the main difference between the groups.

The most common software platforms for metabolomics data analysis are XCMS and Metaboanalyst, which consist of online platforms that integrate statistical analysis for metabolomics data and, also, algorithms that pretreat the data to improve subsequent statistical analyses. Both are public platforms with an intuitive and user-friendly layout that are commonly used in metabolomics studies ( 51 , 52 ).

### Metabolomics database

The metabolomics approach aims to differentiate groups from comparative analyses between samples. In clinical research, the metabolome of the group affected by a given diseases compared to the group considered healthy in order to obtain information on the altered phenotypic profile, to search for biomarkers, for mechanisms elucidations, and physiology of the disease stages ( 53 – 55 ). In this scenario, it is essential to identify the different metabolites in order to look precisely at their cellular pathways and correlate the different metabolites with the physiological difference.

In the case of metabolomics, there are a variety of databases containing predicted and/or real MS and NRM spectra metabolites. The human metabolome database (HMDB) is a good example, that links a range of metabolites annotated by referenced spectral data. From 2007 until 2018, the total number of metabolites increased from 2,180 to 11,410,038 ( 56 ). For the field of metabolomics, the Metlin database is one of the largest and most relevant public repositories of low molecular weight molecules available to date, holding more than 900,000 molecules. It is important to understand that Metlin is a platform that holds a public library and cloud-based data analysis tools that integrate quantitative and statistical data analysis, which promote the use translational and cooperative MS ( 57 )

Each metabolite identification platform has its own distinct characteristics, which must be carefully deliberated by the researchers according to their objective. The two largest studies involving GHD used NMR spectra from HMDB to identify the metabolites. This step is crucial for the correct interpretation of the effects of the differential metabolites in the cellular pathways in patients. It is the final step of any metabolomics work to achieve a complete understanding of the link between the biological chemistry and the physiology of the disease.

### Metabolomics approach in growth hormone deficiency

There are a small number of studies in the specialized literature on the application of metabolomics in GHD research and its application as a tool in clinical practice. Most commonly found are discussions about the increasing importance and application of metabolomics in some results intrinsic to the clinical condition of GHD patients. However, only one of the articles addresses possible biomarkers for diagnosis and/or control of rhGH replacement ( 26 ).

One of the first papers to use metabolomics to evaluate the physiology of deficient GHD was by Hoybye and cols. (2014) ( 26 ). In the experimental design, the authors used serum from 10 adult patients diagnosed with severe GHD; however, they were not all congenital and isolated. Due to the high research capacity to cover the human metabolome, there is a growing interest in applying metabolomics to understand changes in the endocrine system affected by GHD. GC-MS (Gas chromatography–mass spectrometry) needs to change the physical–chemical properties of the metabolites before analyzing the samples, which may result in a decrease in the number of metabolites. The results were interesting due to the changes in metabolites related to lipid and protein class, showed to be the most prominent classes as alternative biomarkers to IGF-1.

Abd Rahman and cols., (2013) applied the metabolomics technique in their study on a single patient diagnosed with GHD, caused by a mutation in the *PIT-1* gene that regulates the expression of GH and other hormones in the pituitary gland. The untargeted approach, using urine as a biofluid and a nuclear magnetic resonance (NMR) analytical tool, showed the impact of rhGH replacement for 5 years, reflecting a decrease in weight and an increase in free fatty acids related to lipolysis stimulation and, consequently, a greater performance of beta-oxidation of lipids ( 27 ). Through NMR it was possible to infer the positive effect of the replacement of GH in the normalization of the metabolic profile, however, even when normalized, the patient's profile remained altered compared to the control group. The research was undertaken with one patient sample and 17 samples as the control group, which led to a statistical deficiency in the comparison, and difficulty interpreting the remaining changes in the metabolic profile of the patient, that could have been driven by alimentary, or any other external factor. However, the results followed the same direction as the first mentioned paper, and showed the lipid pathway as extremely relevant to the development of GHD disease.

Regarding the use of rhGH by individuals without GHD, it is recurrent due to the interest in the metabolic effects caused by this hormone. Thus, understanding rhGH will also benefit from relevant information that goes beyond GH deficient, as a recent study applied the metabolomics technique to the use of rhGH by athletes ( 58 ). The most recent study using metabolomics in GHD research was the metabolomics profile of serum samples from children with short stature was performed by NMR and revealed disturbances in glucose metabolism and amino acid biosynthesis ( 59 ). The study reached 12 possible biomarkers involved in the physiological disorder. This referred article confirmed metabolic differences between a healthy group from children with short stature; however, short stature cohort was represented by idiopathic short stature and GHD. The inclusion of patients with idiopathic short stature may have conditioned analytical suppression which influenced the identification of significant metabolites. In metabolic studies, it is very important that the samples are homogeneous, as this helps to avoid confounding factors and false positives ( 59 ).

It is noticeable that changes in the GH axis are influenced by the stages of human development, however, late diagnosis is common ( 19 – 21 ). Based on the complexity of GHD, the creation of diagnostic signatures through metabolomics must consider metabolites directly affected by the GH axis and how they will be divided according to each stage of life; contributing to rapid diagnoses and better distinction between diseases affected by changes in the GH axis.

## GHD: THE CHALLENGES IN DIAGNOSIS AND CONTROL OF TREATMENT IN THE LIGHT OF CURRENT EVIDENCE

The diagnosis of GHD at both stages of life (children and adult) requires an extensive evaluation that utilizes both biochemical and imaging examinations. IGF1 and IGFB3 are GH action markers, but sometimes they are not helpful for the diagnosis, and stimulus tests for GH releasing are necessary, but sometimes not reproducible. Some studies have been performed to try to find a metabolic signature for GHD, but because of the limited cohort number or even analyzing tools, there is not a signature accepted in the literature for GHD diagnosis and treatment follow up.

Given this discussion, further studies with different biofluids and multiple analytical tools are essential for establishing an efficient diagnostic signature of GHD, as well as for determining alternative biomarkers for monitoring the effects of rhGH therapy and, consequently, improving the approach used for these patients. Once that metabolomics studies could find diferent pathways linked to GHD and significant metabolites in this clinical set, all these knwoledge could be used to explore less invasive techincs and different biofluids in the search for a better diagnostic and prognostic methods. This kind of improvement will be very useful in particular for pediatric population.

## FUTURE PERSPECTIVES

The proposal to expose the fundamentals of metabolomics sciences as a prominent technique in the diagnosis-aid of endocrine diseases claims the creation of a multidisciplinary group that aims to implement omics technology as tools in the diagnosis. To reduce scientific results without application, translational medicine is used in the communication between the areas of knowledge that dominate medical clinic and clinical bioanalytics.

This review paper critically addressed the researchers to enhance the number of papers in this field, and even more importantly, it is necessary to perform these researches with caution, knowledgeable experiments, and including well-thought-out data analysis. This review paper have been focused on critically analyzing the most important steps in metabolomics study, highlighting what we believe to be the best approach for new GHD metabolomics studies.

Given this discussion of findings, it is evident that further studies are essential to establish an efficient diagnostic signature for GHD, as well as to determine alternative biomarkers for monitoring the effects of rhGH therapy and, consequently, improving the clinical approach with these patients. Metabolomics has powerful bioanalytical tools that can make a significant contribution to investigating endocrine diseases through large-scale molecular analyses. In order to enrich the GHD metabolomics studies, it is very important to encourage researchers to enhance the number of papers in this field, and even more importantly, it is necessary to perform these researches with caution, knowledgeable experiments, and including well-thought-out data analysis.

The metabolic axis of GH has not yet been fully elucidated, so the development of omics positively favors more accurate responses. The next steps for omics technologies will probably be to integrate data between genomics, transcriptomics, proteomics, and metabolomics/lipidomics. The associations found among them may lead to a greater understanding of the physiological changes and, consequently, improve GH replacement with adequate doses. In the few studies already applying metabolomics in GHD research, it is evident that the lipid class is affected, and therefore, it seems essential for the as yet unexplored lipidomics to be further investigated.

Recently, some studies have used untargeted and targeted metabolomics in the search for new biomarkers for acromegaly disease; GH overproduction disease has also been shown to affect the lipid profile ( 32 , 44 , 60 ).

Many studies and discoveries that have occurred and continue to occur on laboratory benches are overlooked because there is no practical application for these results. Therefore, even where the research of metabolomics in GHD has presented solid data, there were no results or proposals for clinical feedback to shape or direct future service applications.

There are some translational medicine centers around the world, that are being recognized for the application in the medical setting of products or services developed in the laboratory research space. It is evident that introducing more translational medicine proposals into clinical research will improve communication between different areas of knowledge, and will facilitate turning theoretical findings into practical solutions.
